# Ultrafast Dissociation Dynamics of the Sensitive Explosive Ethylene Glycol Dinitrate

**DOI:** 10.1021/acs.jpclett.4c03220

**Published:** 2025-01-18

**Authors:** Erica Britt, Hugo A. López Peña, Jacob M. Shusterman, Kunjal Sangroula, Ka Un Lao, Katharine Moore Tibbetts

**Affiliations:** Department of Chemistry, Virginia Commonwealth University, Richmond, Virginia 23284, United States

## Abstract

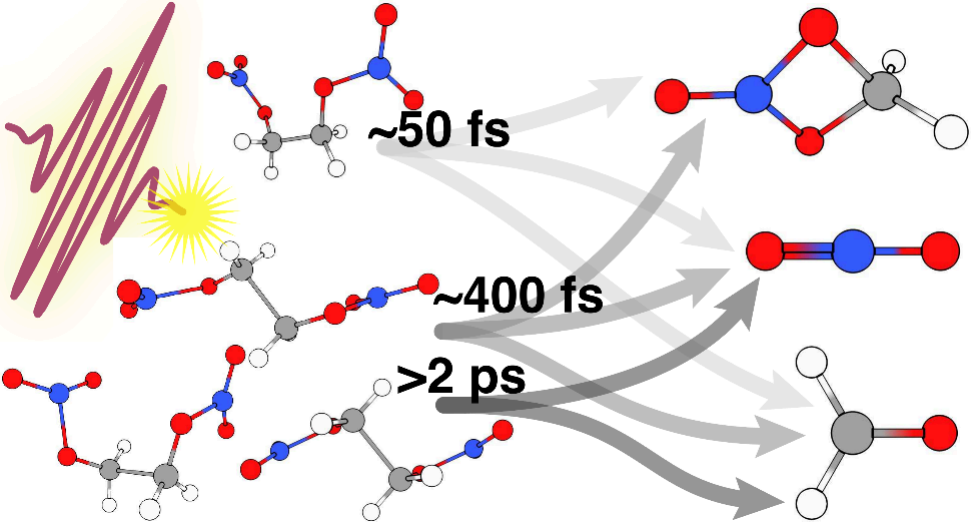

Ethylene glycol dinitrate (EGDN) is a nitrate ester explosive widely used in military ordnance and missile systems. This study investigates the decomposition dynamics of the EGDN cation using a comprehensive approach that combines femtosecond time-resolved mass spectrometry (FTRMS) experiments with *ab initio* electronic structure and molecular dynamics computations. We identify three distinct dissociation time scales for the metastable EGDN cation of approximately 40–60 fs, 340–450 fs, and >2 ps. The observed dissociation time scales are rationalized by electronic and geometric relaxation of multiple EGDN conformers. These insights are crucial for advancing knowledge of the initial molecular decomposition processes that are central to the detonation physics of nitrate esters, which can lead to improving safety protocols and optimizing the performance of nitrate ester explosives in various applications.

Nitrate esters, such as ethylene glycol dinitrate (EGDN), nitroglycerin (NG), erythritol tetranitrate (ETN), pentaerithritol tetranitrate (PETN), and nitrocellulose (NC), are widely used as explosives and propellant ingredients.^1^ The highly labile O–NO_2_ moiety makes nitrate esters particularly sensitive energetic materials.^2^ For instance, EGDN is classified as a primary explosive due to its impact sensitivity of only 0.2 J^1^ (for comparison, the commonly used secondary explosive RDX has an impact sensitivity of 7.4 J^3^). Impact (shock) sensitivity is particularly crucial for military applications because the risk of accidental detonation of explosives directly affects the safety of their handling, transportation, and storage.^4^ Theoretical studies have established that shock initiates electronic excitation, ionization, and electron–ion coupling that induce rapid molecular decomposition in explosives, in part by lowering dissociation barriers.^5−8^ For instance, the PETN cation was found to have a negligible 0.1 kcal/mol barrier to O–NO_2_ bond cleavage.^8^ Therefore, studying nitrate ester cations is important for understanding their initial decomposition processes, which can lead to detonation. However, analysis of nitrate ester decomposition by mass spectrometry is extremely challenging because nitrate esters, including EGDN, NG, and PETN, produce the same major fragment ions and no intact parent molecular ion.^9,10^

To overcome this challenge, we used femtosecond time-resolved mass spectrometry (FTRMS) to interrogate the dissociation dynamics of the EGDN cation (EGDN^+^). FTRMS is a pump–probe technique in which an intense “pump” pulse induces ionization and a time-delayed weak “probe” pulse electronically excites the cation to disrupt spontaneous relaxation or dissociation pathways.^11,12^ FTRMS has been used to determine time scales of molecular relaxation^13,14^ and rearrangement,^15,16^ prepare coherent vibrational states,^17−22^ capture sub-picosecond dissociation time scales of metastable dications,^23−27^ and quantify the compositions of isomer mixtures.^28^ In this work, we combine FTRMS measurements of EGDN with high-level electronic structure and *ab initio* molecular dynamics (AIMD) calculations. We employ the conformer–rotamer ensemble sampling tool (CREST)^29−31^ with the semiempirical quantum chemistry package xtb,^32,33^ density functional theory (DFT) at the B3LYP/6-31G(2df,p) level^34−37^ using Gaussian 16,^38^ along with equation-of-motion coupled-cluster singles and doubles (EOM-CCSD)^39^ using the 6-311+G(d) basis,^40^ and AIMD^41^ at the B3LYP/6-31G(2df,p) level using Q-Chem 5.3.^42^

We measure the dynamics of EGDN^+^ using a 1300 nm, 20 fs pump pulse with an intensity of 10^14^ W cm^–2^ and a 650 nm, 47 fs probe pulse with an intensity of 10^13^ W cm^–2^ (see the Supporting Information for details). Under these conditions, the strong field ionization (SFI) mechanism is in the tunneling regime^43^ and the probe pulse is too weak to induce ionization independently. Figure 1a shows that the mass spectrum of EGDN obtained with only the pump pulse is similar to the spectrum obtained with 70 eV electron-impact ionization (EI) from the National Institute of Standards and Technology (NIST).^9^ Both spectra produce the fragment ions CH_2_NO_3_^+^ (*m*/*z* 76), NO_2_^+^ (*m*/*z* 46), CH_2_O^+^ (*m*/*z* 30), and CHO^+^ (*m*/*z* 29), with no intact molecular ion. The lack of molecular ion detection, even under SFI tunneling conditions, indicates that EGDN^+^ is metastable to dissociation. The change in the mass spectrum when the probe pulse is applied at a delay of +60 fs (Figure 1b) shows the depletion of both NO_2_^+^ and CH_2_NO_3_^+^, with concomitant enhancement of CH_2_O^+^ and CHO^+^. This result indicates that the application of the probe pulse at sufficiently short delay times disrupts the spontaneous SFI-induced dissociation pathways of EGDN^+^.

**Figure 1:**
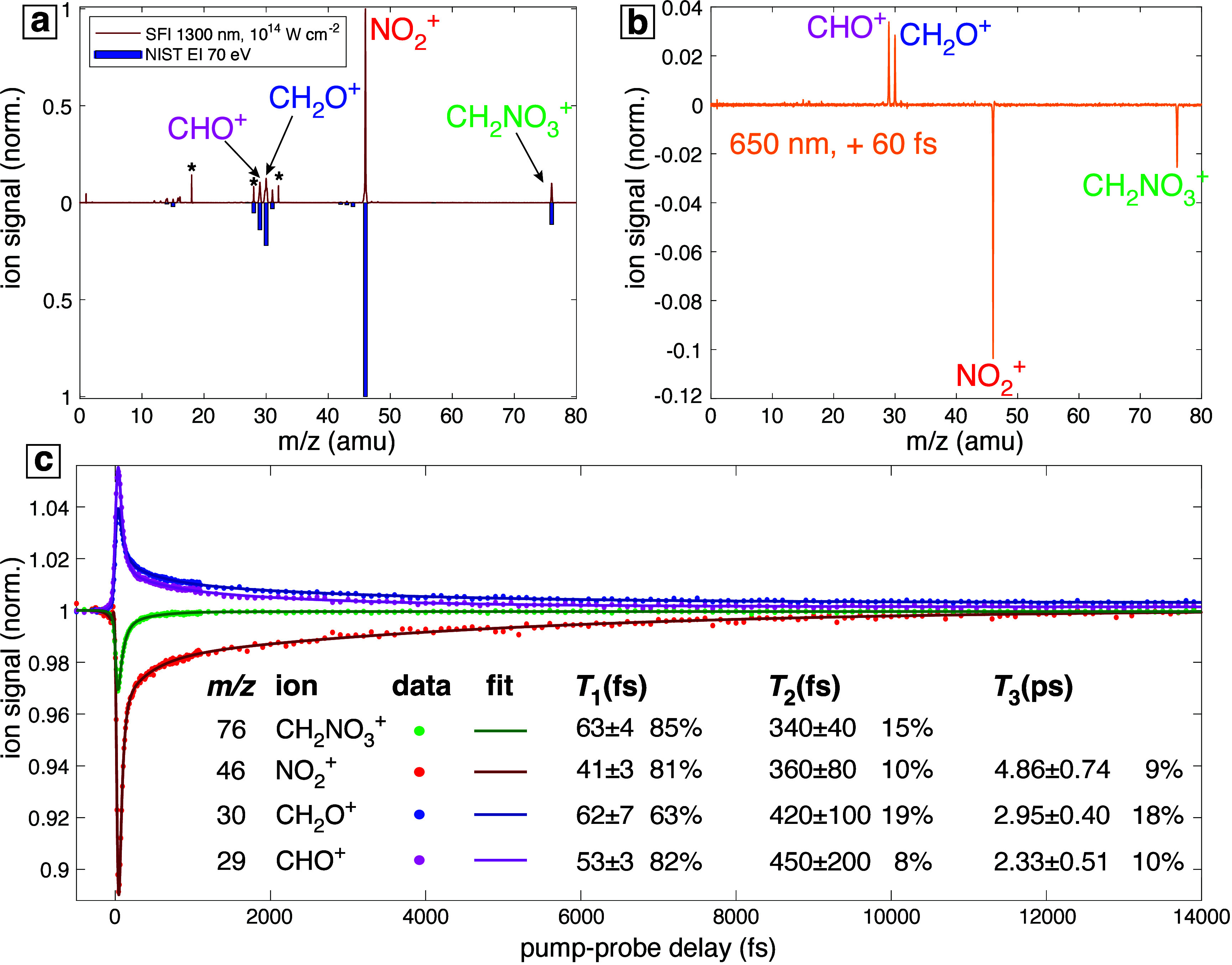
(a) SFI and EI^9^ mass spectra of EGDN with labeled fragment ions. ∗ indicates residual H_2_O^+^, N_2_^+^, and O_2_^+^ from air. (b) Difference mass spectrum obtained with application of the probe pulse at a delay of +60 fs. (c) Transient ion signals of EGDN fragment ions CH_2_NO_3_^+^ (green), NO_2_^+^ (red), CH_2_O^+^ (blue), and CHO^+^ (magenta), with fits to a series of decaying exponential functions convoluted with the instrument response function (see the Supporting Information for details). The time constants and their relative contributions to the dynamics of each ion are indicated.

The transient signals of each fragment ion measured over a pump–probe delay range from −500 fs to +14 ps are shown in Figure 1c. Each ion signal exhibits a transient depletion (CH_2_NO_3_^+^ and NO_2_^+^) or enhancement (CH_2_O^+^ and CHO^+^) and then returns close to its initial value by the end of the 14 ps measurement window. These features resemble the dynamics of fragment ions produced from metastable dications,^23−27^ suggesting that the observed fragment ion dynamics arise from dissociation of the metastable EGDN^+^ ion. The dynamics were fit to a series of exponential decays convoluted with a Gaussian instrument response function (IRF), as described in previous studies^12,13^ (see the Supporting Information for details). The fitting procedure yields three time constants associated with ion dynamics at positive pump–probe delays, as indicated in Figure 1c. The fit coefficients are provided in the Supporting Information. All transients exhibit a fast time constant (*T*_1_ ∼ 41–63 fs), which contributes more than 80% to the observed dynamics in each ion except for CH_2_O^+^ (63%). Minor contributions from a slower sub-picosecond time constant (*T*_2_ ∼ 340–450 fs) were observed for all ions, and an even slower time constant (*T*_3_ ∼ 2–5 ps) was observed for all ions except CH_2_NO_3_^+^.

Close examination of the fragment ion dynamics near pump–probe temporal overlap at zero delay reveals additional features. Figure 2 shows the experimental FTRMS signals (dots) with the complete fit functions (solid lines), along with the fit components (dashed lines) corresponding to the IRF (gray), *T*_1_ (orange), *T*_2_ (dark orange), *T*_3_ (yellow), and *T*_neg_ (light blue). The *T*_neg_ time constant is associated with dynamics at a negative pump–probe delay (i.e., when the probe pulse precedes the pump pulse), where the probe interacts with neutral EGDN. This time constant *T*_neg_ ∼ 50 fs, present in the fitting for all transients, can be associated with dynamics following a three-photon absorption of the 650 nm probe pulse (*vide infra*). The dynamics associated with the fast time constant *T*_1_ were delayed by 15–24 fs relative to zero delay, as indicated by the vertical dotted gray lines at zero and *T*_1_ delays (with shaded orange regions denoting uncertainty in the *T*_1_ delay) in Figure 2. This delayed onset of ion yield dynamics has been associated with rapid electronic relaxation following ionization into a cationic excited state.^13^ Notably, the delays of 24 ± 1 and 21 ± 2 fs associated with NO_2_^+^ and CHO^+^ are longer than the delays of 15 ± 2 and 17 ± 3 fs associated with CH_2_NO_3_^+^ and CH_2_O^+^. Statistical analysis presented in the Supporting Information suggests that the *T*_1_ delay is not strictly necessary to account for the dynamics of CH_2_NO_3_^+^ and CH_2_O^+^; therefore, the dynamics of those ion signals may be considered as instantaneous following ionization. In contrast, the same analysis for NO_2_^+^ and CHO^+^ ions yielded statistical significance at the *p* < 0.0001 level, indicating that the delayed onset of *T*_1_ dynamics is necessary for these ions. These results suggest that (1) the fast dynamics associated with *T*_1_ may be attributed to a direct anticorrelation of CH_2_NO_3_^+^ with CH_2_O^+^ and NO_2_^+^ with CHO^+^ and (2) the EGDN^+^ ions that spontaneously dissociate to form NO_2_^+^ within *T*_1_ ∼ 41 fs are mostly ionized into electronic excited state(s), whereas those that spontaneously dissociate to form CH_2_NO_3_^+^ within *T*_1_ ∼ 63 fs are mostly ionized into the ground electronic state.

**Figure 2:**
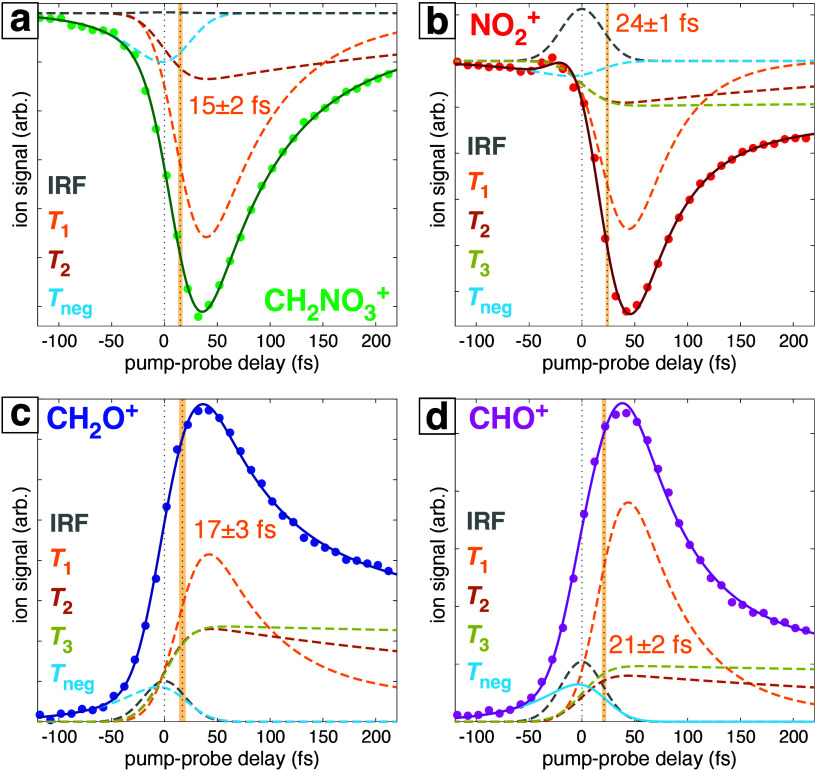
Detail of the transient ion signals of EGDN fragment ions near zero delay showing the different terms contributing to the fitting: IRF (gray), *T*_neg_ (light blue), *T*_1_ (orange), *T*_2_ (dark orange), and *T*_3_ (yellow). Gray dotted lines denote zero delay and the delay corresponding to onset of *T*_1_ dynamics, with the orange shaded region indicating error in the *T*_1_ delay obtained from fitting. Numerical values in orange text denote the delayed onset of *T*_1_ dynamics.

To better understand the experimental dynamics, we conducted detailed electronic structure computations on EGDN and its cation. Details of the computational methods are provided in the Supporting Information. A conformational search using DFT at the B3LYP/6-31G(2df,p) level identified seven neutral conformers (labeled A–G) and four cationic conformers (labeled I^+^–IV^+^), along with their Boltzmann populations and the mapping between vertically ionized neutral conformers and relaxed cation conformers, shown in Figure 3a. EOM-CCSD calculations identified the energies and oscillator strengths of electronic excited states in selected EGDN neutral and cation conformers. The EOM-CCSD calculations were benchmarked to the experimental absorption spectrum of EGDN;^44^ the calculated neutral excited state energies were within 0.6 eV of the center of a prominent absorption feature (see the Supporting Information for details). Figure 3b shows the electronic excited state energies and corresponding oscillator strengths of selected transitions out of the D_0_ state (*f*_0*N*_, where *N* indicates the final state) for EGDN^+^ at the geometry of neutral conformer B (B_vert_^+^, left) and the corresponding relaxed cation conformer II^+^ (right). The energy difference of ∼1.24 eV between D_0_ for the B_vert_^+^ and II^+^ geometries can be considered as excess internal energy or relaxation energy capable of driving fragmentation processes. This relaxation energy exceeds the calculated energy needed for fragmentation of EGDN^+^ into NO_2_^+^ (0.33 eV) and CH_2_NO_3_^+^ (0.36 eV), and it is almost equal to the fragmentation energy for CH_2_O^+^ (1.30 eV), as indicated by the dashed lines in Figure 3b. The high internal energy of vertically ionized EGDN^+^ relative to the low dissociation barriers confirms that EGDN^+^ is metastable upon vertical ionization and accounts for its absence in our FTRMS measurements.

**Figure 3:**
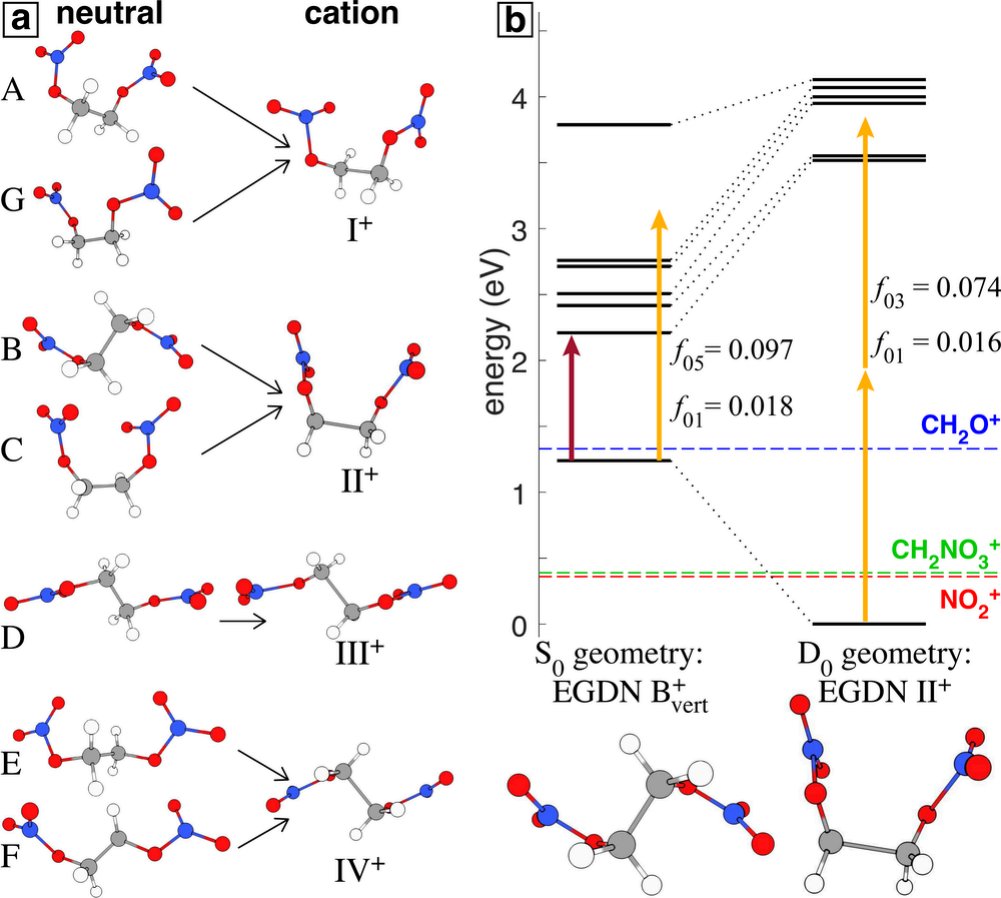
(a) Mapping between EGDN neutral and cation conformers upon geometric relaxation after vertical ionization. (b) Electronic excited state energies and corresponding oscillator strength (*f*) of selected transitions out of the D_0_ state for B_vert_^+^ (left) and II^+^ (right) calculated at the EOM-CCSD/6-311+G(d) level of theory. The red and orange arrows represent the photon energies of the 1300 nm pump and 650 nm probe pulses, respectively.

The EOM-CCSD calculations also provide insights into the electronic excited states that could contribute to the observed experimental dynamics. The 1300 nm (0.95 eV) pump pulse (red arrow in Figure 3b) has sufficient energy to ionize EGDN into the D_1_ state of B_vert_^+^ through absorption of an excess photon due to the high *f*_01_ oscillator strength. As a result, it is expected that some population of EGDN molecules is ionized directly into the D_1_ state. Therefore, the previously discussed delayed onset of 15–24 fs for the fast *T*_1_ decay may be attributed to electronic relaxation of EGDN^+^ from D_1_ to D_0_, similar to the dynamics observed in the ethylene cation.^13^ The probe pulse at 650 nm (1.91 eV, orange arrow in Figure 3b) is capable of populating the D_1_–D_5_ excited states of B_vert_^+^ through one-photon absorption. After relaxation to conformer II^+^, the D_1_ state is nearly 3.5 eV above the ground state, precluding a one-photon transition. We hypothesize that a two-photon transition could populate D_1_ or D_2_, as illustrated in Figure 3b. Additionally, a strong S_0_ to S_4_ transition with excitation energy around 6 eV was identified in neutral conformers B and G (see the Supporting Information), which could be reached by three-photon absorption of the 650 nm probe (5.72 eV). Therefore, the observed *T*_neg_ ∼ 50 fs time constant discussed above can be associated with three-photon excitation to S_4_ and ionization by the pump pulse within the ∼50 fs lifetime of the S_4_ state.

To assess how the three observed time constants at positive pump–probe delay are related to the dissociation of metastable EGDN^+^, we performed AIMD simulations to model the dynamics of the EGDN cation (see the Supporting Information for details). Simulations were initiated from the vertically ionized structure of conformer G (G_vert_^+^) to model the dynamics immediately after vertical ionization and from the cation conformers I^+^–IV^+^ to model the dynamics of the relaxed cation. To consider the relaxation energy of these conformers resulting from vertical ionization, we put zero-point vibrational energy (ZPE) into each normal mode to select the initial nuclear velocities (combined with randomized signs for the velocity components). This initialization method is reasonable considering that AIMD trajectories propagate on top of the Born–Oppenheimer surface (without ZPE) and that the ZPE of ∼2.33 eV for all relaxed cation conformers exceeds the relaxation energies in the range of 1.12 and 1.33 eV. The same initialization method was used for the trajectories starting from G_vert_^+^. To determine the lifetime of EGDN^+^ prior to dissociation, we defined the initial fragmentation time (IFT) as the time corresponding to the first bond-breaking event. A bond-breaking event was identified when the distance between two atoms exceeded the equilibrium bond length by at least 50%.

The IFT statistics for simulations initiated from each conformer are summarized as a box plot in Figure 4, including the percentage of trajectories with at least one bond breakage within the 1100 fs simulation time. Complete distributions of IFTs for each conformer are given in the Supporting Information. G_vert_^+^ was found to dissociate in all trajectories and most rapidly with a mean IFT of 100 fs and median IFT of 85 fs, similar to the experimental *T*_1_ ∼ 41–63 fs (Figure 1c). We note that the slightly faster experimental *T*_1_ could arise from additional energy absorbed upon ionization into the electronically excited D_1_ state, as discussed above. In contrast, the mean IFT values for conformers I^+^–IV^+^ ranged from 322 to 607 fs, roughly comparable to the experimental *T*_2_ ∼ 340–450 fs (Figure 1c). Moreover, some trajectories for conformers I^+^, III^+^, and IV^+^ did not dissociate within the simulation window. This result suggests that some proportion of these conformers dissociate on a slower time scale, which can account for *T*_3_ ∼ 2–4 ps observed in the NO_2_^+^, CH_2_O^+^, and CHO^+^ fragments in Figure 1c.

**Figure 4:**
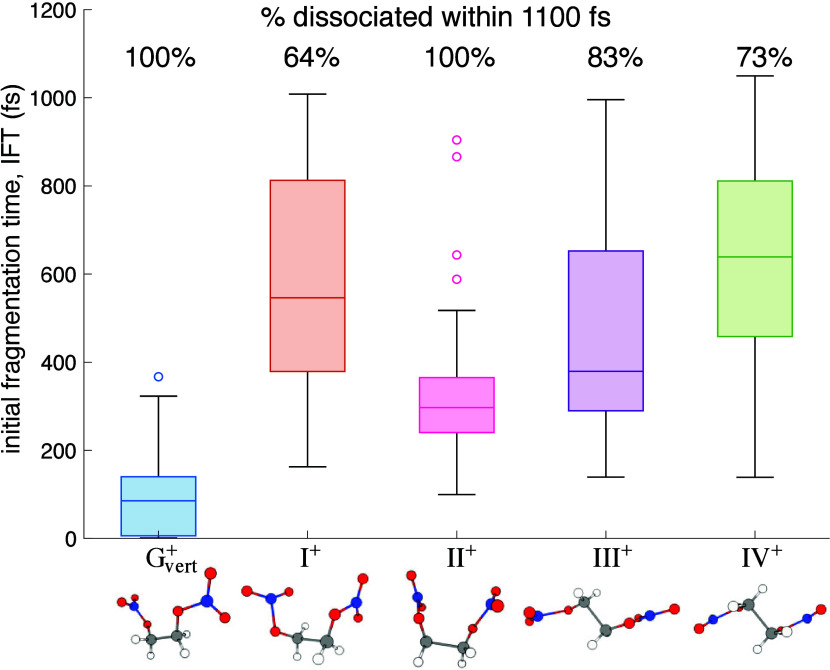
Box plot of the IFT dsitributions for conformers G_vert_^+^ and I^+^–IV^+^, with the percentage of trajectories with at least one bond-breaking event indicated for each conformer.

Statistical analysis of the differences among EGDN^+^ conformers in IFT and fragmentation probability presented in the Supporting Information consistently grouped them into three categories: (1) conformers I^+^, III^+^, and IV^+^, which take ∼470–610 fs to dissociate and have non-zero probability of remaining intact at the end of the simulation time, (2) conformer II^+^, which dissociates with 100% probability significantly faster (∼320 fs) than the I^+^/III^+^/IV^+^ group, and (3) G_vert_^+^, which dissociates with 100% probability significantly faster (∼100 fs) than conformer II^+^. This grouping allows us to assign the observed experimental time constants to fragmentation processes based on initial conformer structures as follows: *T*_1_ ∼ 41–63 fs can be assigned to direct dissociation of EGDN^+^ immediately after vertical ionization, before geometric relaxation to one of the cation conformers. On the basis of the contribution of *T*_1_ to the experimental dynamics, it is expected that ∼80% of ionized EGDN molecules dissociate before geometric relaxation. The remaining ∼20% of EGDN molecules can relax to any cation conformer and dissociate on the *T*_2_ ∼ 340–450 fs time scale, with a 100% dissociation probability expected for only conformer II^+^. Finally, some fraction of the EGDN molecules that relax to I^+^, III^+^, or IV^+^ can survive intact for longer, dissociating on the *T*_3_ ∼ 2–5 ps time scale.

This work reports the first, to our knowledge, direct experimental measurement of sub-picosecond dissociation time scales in an active nitrate ester explosive, EGDN. The experimental observation of three distinct dissociation time scales in the EGDN cation through FTRMS measurements is readily explained by the theoretical results indicating contributions from electronic and geometric relaxation following vertical ionization, along with the presence of multiple EGDN conformer structures. These findings are significant for advancing knowledge of molecular-scale behavior in nitrate ester explosive materials, such as EGDN, NG, and PETN, which are widely used in military and industrial applications. Ultimately, this work contributes to the broader goal of enhancing safety in handling and developing more efficient energetic materials while also providing insight into the complex chemical processes that occur during detonation.

## Supporting Information Available

Experimental and theoretical methods, characterization of synthesized EGDN, characterization of the instrument response function, tabulated coefficients of curve fitting and statistical analysis for experimental data, tabulated computational results: IE values, geometric parameters, and Boltzmann population analysis, relaxation energies, excited state energies, and oscillator strengths, statistical analysis of AIMD trajectories, graphical computational results: conformer structures and excited electronic states, comparison of computed excited state energies to experimental absorption spectrum, dissociation pathways, and AIMD simulation results (PDF)
