# Supplementary material for: Ultrafast Dissociation Dynamics of the Sensitive Explosive Ethylene Glycol Dinitrate
Source: J Phys Chem Lett. 2025 Jan 18;16(4):882–8. doi: 10.1021/acs.jpclett.4c03220 (PMC11789135; doi:10.1021/acs.jpclett.4c03220)
Supplement: Supplementary file 1 — jz4c03220_si_001.pdf [file jz4c03220_si_001.pdf]

Supporting Information: Ultrafast Dissociation Dynamics of the Sensitive Explosive Ethylene Glycol Dinitrate (EGDN)

Erica Britt, Hugo A. López Peña, Jacob M. Shusterman, Kunjal Sangroula, Ka Un Lao, and Katharine Moore Tibbetts*

Department of Chemistry, Virginia Commonwealth University, Richmond, VA 23284, United States

E-mail: kmtibbetts@vcu.edu

Contents

Number of pages: 31

Number of figures: 10

Number of tables: 19

S1 Experimental methods and data analysis

S1.1 EGDN synthesis

EGDN, a nitrate ester, is a colorless, oily, syrupy liquid with a melting point of –22.8 °C. It detonates when heated to over 70 °C. EGDN was produced according to the following reaction:

$$C_2H_6O_2 + 2\,HNO_3 \longrightarrow C_2H_4(NO_3)_2 + 2\,H_2O$$

EGDN was prepared by mixing a nitric and sulfuric acid mixture with ethylene glycol. The nitrated compound forms quite readily and is easily recovered by drowning the entire reaction mixture into cold water, followed by separating the EGDN layer, washing, and then drying. In this study, EGDN was synthesized from laboratory-grade ethylene glycol (EG.Pur) provided by Fluka Chemika. We used a mixed acid nitration with sulfuric acid (98%) and concentrated nitric acid (68%). Post-synthesis, the product underwent a brief wash with distilled water, followed by washing with aqueous sodium carbonate solution to neutralize residual acids, and further washing with distilled water until reaching a neutral pH.[S1] The product yield was approximately 70%. The successful synthesis of ethylene glycol dinitrate (EGDN) was confirmed using gas chromatography-mass spectrometry (GC-MS) analysis performed on an Agilent 6890N GC coupled with a 5973 MSD using an electron impact (EI) source. The instrument was equipped with a DB-1MS capillary column (30 m length, 0.2500 mm internal diameter), which provided effective separation and identification of the synthesized EGDN (Figure S1).

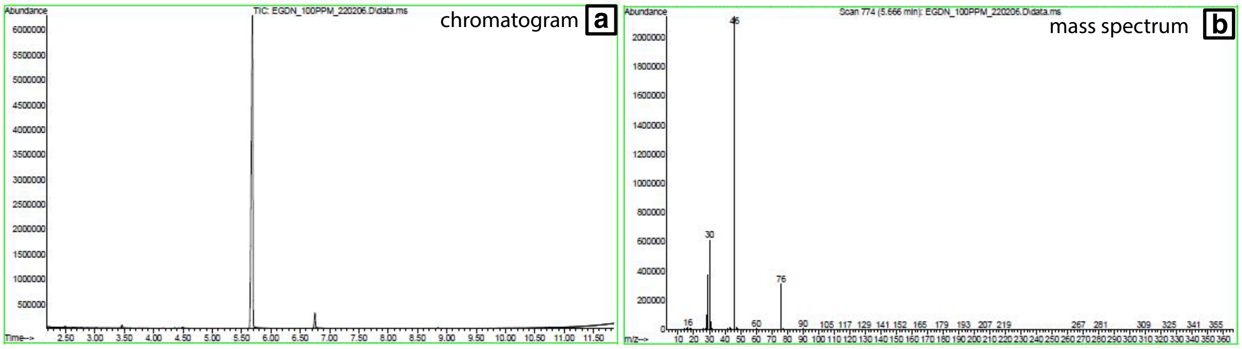

Figure S1: GC-MS analysis confirming the successful synthesis of ethylene glycol dinitrate (EGDN). The chromatogram (a) shows a distinct peak corresponding to EGDN (at 5.76 min) with the mass spectrum (b) displaying characteristic fragmentation patterns (30 m/z, 46 m/z, and 76 m/z) consistent with the EGDN molecular structure.[S2]

S1.2 Instrumentation

The instrumentation has been described in detail in our previous work.[S3] Briefly, 2.2 mJ of output from a commercial Ti:Sapphire regenerative amplifier (Coherent Astrella, producing 800 nm, 30 fs pulses at 1 kHz repetition rate) was used to pump an optical parametric amplifier (OPA, Topas Prime). The OPA output at 1300 nm signal wavelength was split with a 50:50 (R:T) beam splitter to produce "pump" and "probe" beam lines. The 1300 nm output was used as the pump pulse for strong-field ionization (SFI). The probe beam was frequency-doubled with a β-barium borate (BBO) crystal to produce 650 nm for optical excitation of ions created by the SFI pump pulse. The pump and probe beams were combined using a dichroic beam splitter and focused into the extraction region of a linear time-of-flight mass spectrometer (TOF-MS) with a f = 20 cm fused silica lens. The dehydrated EGDN sample was introduced into the TOF-MS vacuum chamber through an effusive source; prior to TOF-MS measurements the sample was exposed to vacuum for 24 hours to remove residual water in the sample. Mass spectra were recorded at a working EGDN pressure of 3×10^{-7} Torr with a 1 GHz oscilloscope (LeCroy WaveRunner 610Zi). The reported transient ion signals were obtained from combining two sets of measurements: (1) measurements collected over the delay range -300 fs to +1100 fs in steps of 10 fs, and (2) measurements collected over the delay range -1000 fs to +14000 fs in steps of 100 fs. The mass spectral data for each pump-probe delay was collected for 50,000 laser shots.

S1.3 Determination of strong field ionization regime

At the intensity of 10^{14} W cm^{-2} used in the experiments, the ionization is dominated by electron tunneling. The Keldysh parameter γ is given by[S4]

$$\gamma = \frac{\omega\sqrt{2m_e I_p}}{eE_0} \tag{S.1}$$

where ω is the angular frequency of the laser field, m_e is the electron mass, I_p is the ionization potential of the molecule, e is the elementary charge, and E_0 is the peak electric field of the laser. When $\gamma < 1$, the molecule ionizes via tunneling ionization (quasi-static regime). The field is strong enough that the electron can tunnel through the potential barrier created by the combined effect of the Coulomb potential and the laser field. If $\gamma > 1$, ionization occurs via multi-photon absorption (perturbative regime). The field is not strong enough to distort the Coulomb potential significantly, so the electron absorbs multiple photons to gain enough energy to ionize. Finally, if $\gamma \approx 1$, the ionization regime is intermediate, involving both tunneling and multi-photon characteristics.[S5] Table S1 lists the values of γ at different wavelengths considering a laser intensity of 10^{14} W/cm^2 and at a calculated vertical I_p of 10.95 eV (see Table S5). At the 1300 nm wavelength used in this study, the value $\gamma \sim 0.6$ indicates that ionization is primarily in the tunneling regime.

Table S1: Keldysh parameters (γ) at different wavelengths considering a laser intensity of 10^{14} W cm^{-2} and a vertical I_p of 10.95 eV.

**Wavelength (nm)**	**Keldysh (γ)**
800	0.962
1200	0.641
1300	0.592
1400	0.550
1500	0.513
2000	0.350

S1.4 Pump-probe data analysis

To establish the instrument response function (IRF) and the zero-delay temporal overlap between the pump and probe pulses, the sum of the impurity ion signals (H_2O^+, ${N_2}^+$, and ${O_2}^+$) was used (Figure S2). Fitting this data to a Gaussian function gave a FWHM of 47 fs, with width parameter $s = 28$ fs used for fitting of the transient ion signals.

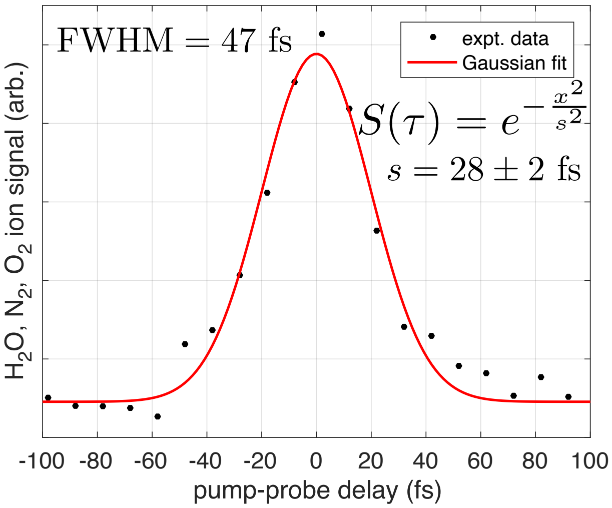

Figure S2: Sum of the transient ion signals of H_2O^+, $N_2{}^+$, and $O_2{}^+$ (dots), with data fit to a Gaussian function (solid red line).

The transient ion signals as a function of pump-probe delay τ were fit to a series of exponential decay functions convoluted with the Gaussian IRF, following literature methods.[S6,S7] The overall fit equation consists of six terms:

$$S(\tau) = ae^{-\tau^2/s^2} + bP(\tau, t, T_1) + cP(\tau, T_2) + dP(\tau, T_3) + f\left(1 + \text{erf}\left(\frac{\tau}{s}\right)\right) + gP(\tau, T_{\text{neg}}) + 1 \quad \text{(S.2)}$$

where $s = 28$ fs was obtained from Figure S2. The constants a, b, c, d, f, g, T_1 T_2, T_3, T_{neg}, and t are variable parameters optimized by nonlinear least squares curve fitting in MATLAB.

The first term in eq S.2 simulates the IRF from the cross-correlation signal (Figure S2). The remaining terms in eq S.2 are associated with the dynamics of the transient ion signals produced from EGDN. The term $P(\tau, t, T_1)$ corresponds to the fastest decay time at positive delay ($\tau \geq 0$) and is given by

$$P(\tau, t, T_1) = \left[1 + \text{erf}\left(\frac{(\tau - t)}{s} - \frac{s}{2T_1}\right)\right] e^{-(\tau - t)/T_1} \quad \text{(S.3)}$$

where the variable parameter t represents a temporally delayed onset of the dynamics, as was used in ref. S7. The terms $P(\tau, T_i)$, $i = 2$, 3, correspond to the slower decay dynamics

of the transient EGDN ion signals and are given by

$$P(\tau, T_i) = \left[1 + \text{erf}\left(\frac{\tau}{s} - \frac{s}{2T_i}\right)\right] e^{-\tau/T_i}. \tag{S.4}$$

The fifth term in eq S.2 accounts for depletion or enhancement of an ion signal as $\tau \to \infty$ relative to its yield at $\tau < 0$. The sixth term $P(\tau, T_{\text{neg}})$ is given by

$$P(\tau, T_{\text{neg}}) = \left[1 - \text{erf}\left(\frac{\tau}{s} - \frac{s}{2T_{\text{neg}}}\right)\right] e^{\tau/T_{\text{neg}}} \tag{S.5}$$

and represents dynamics of EGDN at $\tau < 0$. These dynamics can be attributed to 3-photon excitation of neutral EGDN by the 650 nm probe pulse (see Section S2.2 below and main text for further details).

The ion signals NO_2^+, CH_2O^+, and CHO^+ required three exponential decays to fit the data (the T_1, T_2, and T_3 terms), whereas the $CH_2NO_3^+$ signal only required the T_1 and T_2 terms. The T_1 term for all ion signals was fit with a variable onset delay t as in eq S.3; eliminating this fit parameter (and using eq S.4 for the T_1 term) worsened the fit quality. Figure S3 illustrates the improvement in fit quality in the region near zero delay when allowing for variability of t for each of the four ion signals. The fits obtained with variable t are shown as the colored curves; eliminating t resulted in the grey curves. The sum of squares due to error (SSE) values for each fit are shown in each panel. Visually, the differences in the fit functions are much more pronounced for the NO_2^+ and CHO^+ ion signals.

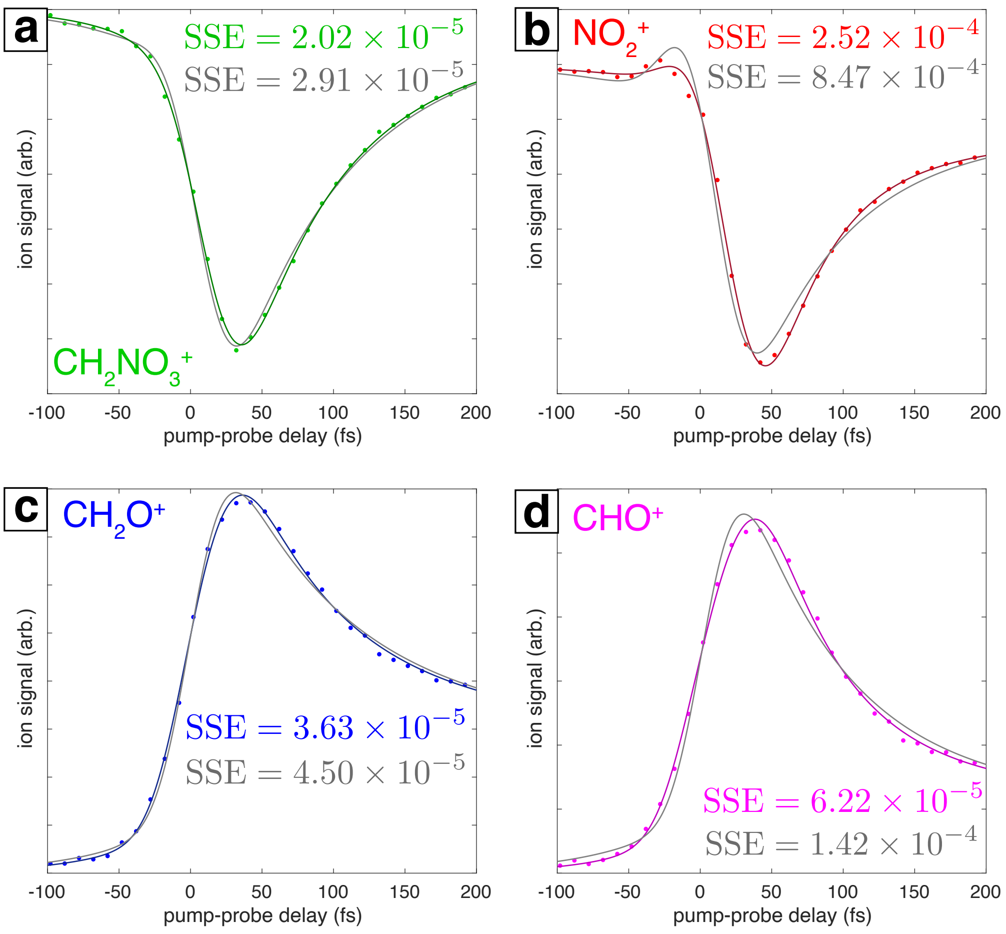

Figure S3: Fit functions to eq S.2 for $CH_2NO_3^+$ (a), NO_2^+ (b), CH_2O^+ (c), and CHO^+ (d), obtained using the variable t parameter (colored curves) and without the t parameter (grey curves).

The fitting parameters for the fits to eq S.2 including the delay t (colored curves in Figure S3) are given in Table S2 and the parameters for the fits exluding t are given in Table S3. The extracted t parameters are significantly larger for NO_2^+ (24 ± 1 fs) and CHO^+ (21 ± 2 fs) than for $CH_2NO_3^+$ (15 ± 2 fs) or CH_2O^+ (17 ± 3 fs). Elimination of the parameter t results in increases to the T_1 values for all ion signals, and large increases in the fitting errors of T_2 and T_3 for the NO_2^+ and CHO^+ ion signals in particular.

Table S2: Fit coefficients to eq S.2, including variable delay t.

Coeff.	$CH_2NO_3^+$	NO_2^+	CH_2O^+	CHO^+
a	0.0001±0.001	0.023±0.004	0.005±0.002	0.010±0.003
b	-0.0202±0.0009	-0.086±0.003	0.0161±0.0008	0.036±0.001
t (fs)	15±2	24±1	17±3	21±2
T_1 (fs)	63±4	41±3	62±7	53±3
c	-0.0036±0.0005	-0.011±0.001	0.0048±0.0005	0.0037±0.0007
T_2 (fs)	340±40	360±80	400±100	450±200
d		-0.0100±0.0005	0.0045±0.0005	0.004±0.001
T_3 (ps)		4.86±0.74	2.95±0.40	2.33±0.51
f	-0.0002	0.0002±0.0004	0.0015±0.0001	0.00069±0.00008
g	-0.004±0.001	-0.005±0.003	0.003±0.001	0.005±0.002
T_{neg} (fs)	43±10	60±27	49±16	45±15

Table S3: Fit coefficients to eq S.2 without variable delay t.

Coeff.	$CH_2NO_3^+$	NO_2^+	CH_2O^+	CHO^+
a	0.009±0.002	0.071±0.008	-0.003±0.002	-0.010±0.003
b	-0.0224±0.0007	-0.082±0.004	0.0174±0.0008	0.038±0.002
T_1 (fs)	76±4	64±4	77±7	70±4
c	-0.0025±0.0006	-0.007±0.002	0.0041±0.0006	0.003±0.004
T_2 (fs)	430±70	740±390	500±170	800±900
d		-0.009±0.001	0.0044±0.0006	0.004±0.004
T_3 (ps)		6.5±4.0	3.02±0.54	2.5±2.0
f	-0.0002	0.0008±0.004	0.0015±0.0001	0.0007±0.0002
g	-0.004±0.001	-0.009±0.005	0.003±0.001	0.005±0.002
T_{neg} (fs)	49±11	60±27	56±16	57±18

To quantify the significance of the delay t to the fit quality, the statistical F-test[S8] was performed, given by the equation

$$F = \left(\frac{\text{SSE}_1 - \text{SSE}_2}{\text{df}_1 - \text{df}_2}\right)\left(\frac{\text{df}_2}{\text{SSE}_2}\right), \tag{S.6}$$

where SSE_i is the sum of squares due to error and df_i is the number of degrees of freedom, where $i = 1$ denotes the fit equation with fewer parameters. For the ion signals ${NO_2}^+$, CH_2O^+, and CHO^+ that require three exponential decays, $\text{df}_1 = 10$ and $\text{df}_2 = 11$; for ${CH_2NO_3}^+$ with two exponential decays, $\text{df}_1 = 8$ and $\text{df}_2 = 9$. The resulting statistics for each ion signal are summarized in Table S4. The p values obtained for the ${NO_2}^+$ and

CHO^+ signals are highly significant ($p < 0.0001$), whereas the p values for the $CH_2NO_3{}^+$ and CH_2O^+ signals are of marginal significance. Hence, the delayed onset of dynamics for the fast T_1 decay is definitely required for interpretation of the $NO_2{}^+$ and CHO^+ dynamics. However, the inclusion of delayed onset dynamics may not be necessary for interpretation of the $CH_2NO_3{}^+$ and CH_2O^+ dynamics, as discussed in the main text.

Table S4: F-test results.

Coeff.	$CH_2NO_3^+$	NO_2^+	CH_2O^+	CHO^+
SSE_1	2.91×10^{-5}	8.47×10^{-4}	4.50×10^{-5}	1.42×10^{-4}
SSE_2	2.02×10^{-5}	2.52×10^{-4}	3.63×10^{-5}	6.22×10^{-5}
df_1	8	10	10	10
df_2	9	11	11	11
F value	3.96	25.9	2.66	14.1
p value	0.028	3.2×10^{-6}	0.062	6.9×10^{-5}

S2 Computational methods

S2.1 DFT calculations

The inherent flexibility of nitrate esters opens the possibility of thermally populating multiple low-energy conformers. Therefore, carefully evaluating these low-energy conformers is relevant for this kind of system. With this idea in mind, we generated ground-state conformational ensembles using the conformer-rotamer ensemble sampling tool (CREST) version 2.11.1 developed by the Grimme group.[S9–S11] CREST was developed as a utility and driver program for the semiempirical quantum chemistry package xtb, also developed by the Grimme group.[S12] The xtb version employed in this work is 6.4.1. CREST uses an iterative meta-dynamics genetic structure crossing (iMTD-GC) workflow with geometry optimization at the GFN2 level, this later method falls into the semiempirical extended tight-binding (xTB) family of methods.[S13] We applied this methodology to both the neutral and cationic EGDN species and to the neutral and cationic fragments generated by dissociation reactions. Conformational ensembles were generated with an energetic threshold of 2

kcal/mol and this yielded nine and six neutral and cationic EGDN conformers respectively. Then the Gaussian 16 software[S14] was used to reoptimize the obtained conformers considering different levels of theory found in literature.[S15,S16] We explored numerous approaches to retain intact optimized structures, including composite methods like PBEh-3c and combination methods such as B3LYP/6-31G*, B3LYP/Def2TZVPP, CAM-B3LYP/6-31G, and B3LYP-6311++G(3df,2p).[S17] For the composite method PBEh-3c, we utilized ORCA 5.0.3.

Table S5: Comparison of vertical and adiabatic ionization energies for EGDN computed at six levels of theory. ND: not computed because neutral EGDN structure fragmented upon ionization.

**Level of theory**	$\mathbf{IE_{ver}}$	$\mathbf{IE_{ad}}$
B3LYP/6-31G*	10.97	9.6
B3LYP/Def2TZVPP	11.19	9.9
CAM-B3LYP/6-31G	11.67	10.61
PBEh-3c	ND	10.69
"B3LYP/6-311++G(3df,2p)"	11.23	9.98
"B3LYP/6-31G(2df,p)"	10.95	9.71

We calculated both vertical and adiabatic ionization energies for EGDN at different theory levels for comparison (Table S5). Due to limited literature on EGDN's specific electronic properties, we compared the calculated vertical ionization energy results to nitromethane, another organic nitro compound, to validate our approach. Based on established chemical principles, we hypothesized that the addition of a second nitrate group in EGDN would likely lower the vertical ionization energy compared to a mono-nitrate compound due to increased electron delocalization. This hypothesis is derived from general understanding of electron-withdrawing groups and their effects on molecular electronic properties. However, we acknowledge that the structural differences between EGDN and nitromethane may limit the accuracy of this comparison. Considering these factors, we estimated the vertical ionization energy of EGDN to be slightly lower than that of nitromethane. Using the previously reported vertical ionization energy of nitromethane, 11.1 eV,[S18] we expected the vertical ionization energy of EGDN to be in the range of $\sim$ 10.8–10.9 eV. This estimate is comparable to our calculated values in Table S5. Through extensive testing and comparisons of geo-

metric parameters such as bond lengths and angles at the B3LYP level with different basis sets, we obtained the following results for neutral conformer B using the 6-31G(2df,p) basis (lengths in Å, angles in degrees): O−N length of 1.42 Å, O=N length of 1.20 Å, C−O−N angle of 114.4°, C−C−O angle of 113.0°, $O{-}N{-}O_{(c)}$ angle of 117.5°, and $O{-}N{-}O_{(t)}$ angle of 112.5°, where 'c' and 't' denote cis and trans conformations, respectively. Additionally, we calculated the bond dissociation energy (BDE) for the $O{-}NO_2$ bond at 149.5 kJ/mol. These values align well with those reported for EGDN in the literature.[S19,S20] To select the appropriate level of theory for subsequent calculations, we ensured that the molecule remained intact and did not dissociate when optimizing the geometries of neutral and cationic species. This analysis determined that DFT at the B3LYP/6-31G(2df,p) level[S19,S21] is most appropriate for EGDN. This conclusion is consistent with findings in other studies of nitro-organic molecules.[S22,S23]

Subsequently, we re-optimized the neutral and cationic conformers, as well as their respective fragments, at this B3LYP/6-31G(2df,p) level. Our analysis included calculations of zero-point vibrational energy (ZPVE), thermal corrections to internal energy, and other thermodynamic properties, allowing for a comprehensive evaluation of the EGDN conformers and their fragments in both neutral and cationic states. Following re-optimization, we applied criteria to distinguish between identical structures, conformers, and rotamers, guided by literature and structural accuracy comparisons.[S10,S15,S24] The criteria included: (1) Root-mean-square deviation (RMSD) between pairs of structures, (2) Rotational constants, and (3) Energetic differences. This process reduced the number of unique conformers to seven neutral and four cationic conformers. The resulting geometric coordinates for the seven neutral and four cationic EGDN conformers are given in Tables S6 through S10.

Table S6: Optimized XYZ coordinates for neutral EGDN conformers A, B, and C at the B3LYP/6-31G(2df,p) level of theory

	Conf. A			Conf. B			Conf. C		
Atom	X	Y	Z	X	Y	Z	X	Y	Z
C	0.5901	-1.5191	-0.5075	0.4105	0.8452	-0.6409	0.7181	0.2568	1.6623
C	-0.5463	-1.4465	0.503	-0.4105	0.8453	0.6409	-0.7181	-0.2568	1.6623
O	1.0399	-0.2297	-0.9462	1.2615	-0.306	-0.7628	1.5855	-0.4202	0.7412
O	-1.726	-0.8755	-0.0823	-1.2615	-0.306	0.7628	-1.5855	0.4202	0.7412
N	-1.8726	0.534	0.1032	-2.4671	-0.2225	0.0161	-1.6806	-0.171	-0.5509
N	1.9111	0.4296	-0.0297	2.4671	-0.2225	-0.0161	1.6806	0.171	-0.5509
O	-2.8365	0.9579	-0.4643	-3.1561	-1.1913	0.1497	-1.0273	-1.1644	-0.7623
O	-1.0598	1.1021	0.789	-2.6498	0.7783	-0.6384	-2.4343	0.4242	-1.2629
O	2.241	1.5113	-0.4161	2.6498	0.7783	0.6384	1.0273	1.1644	-0.7623
O	2.2073	-0.1649	0.979	3.1561	-1.1914	-0.1497	2.4343	-0.4242	-1.2629
H	0.2436	-1.9827	-1.4346	1.0016	1.7618	-0.7002	1.1678	0.0293	2.6335
H	1.4227	-2.0961	-0.0984	-0.2332	0.7731	-1.5189	0.7483	1.3346	1.4934
H	-0.2615	-0.8962	1.3984	-1.0016	1.7618	0.7002	-1.1678	-0.0293	2.6335
H	-0.8467	-2.4611	0.7814	0.2332	0.7731	1.5189	-0.7483	-1.3346	1.4934

Table S7: Optimized XYZ coordinates for neutral EGDN conformers D, E, and F at the B3LYP/6-31G(2df,p) level of theory

	Conf. D			Conf. E			Conf. F		
Atom	X	Y	Z	X	Y	Z	X	Y	Z
C	0.3805	0.2632	0.6092	0.4102	-0.6728	-0.6458	0.4179	0.0531	0.3468
C	-0.3805	-0.2632	-0.6092	-0.4102	-0.6728	0.6458	-0.6619	1.0785	0.0064
O	1.6245	0.8611	0.2151	1.8002	-0.9044	-0.3722	1.6215	0.6178	-0.1942
O	-1.6245	-0.8611	-0.2151	-1.8002	-0.9044	0.3722	-1.9109	0.7174	0.611
N	-2.6898	0.066	-0.0207	-2.5295	0.2612	-0.0066	-2.6741	-0.2322	-0.1338
N	2.6898	-0.066	0.0207	2.5295	0.2612	0.0066	2.7675	-0.199	0.0002
O	-3.7088	-0.4752	0.2953	-3.6859	0.0252	-0.2045	-2.202	-0.6381	-1.1678
O	-2.4481	1.2369	-0.1921	-1.9195	1.3	-0.0788	-3.7121	-0.4935	0.3998
O	2.4481	-1.2369	0.1921	1.9195	1.3	0.0789	3.7612	0.2895	-0.4561
O	3.7089	0.4751	-0.2953	3.6859	0.0252	0.2045	2.6026	-1.2438	0.5804
H	0.5492	-0.531	1.3377	0.2802	0.2516	-1.2089	0.21	-0.9136	-0.1158
H	-0.158	1.0897	1.0765	0.1285	-1.5281	-1.2651	0.5071	-0.0747	1.4292
H	0.158	-1.0897	-1.0765	-0.2802	0.2516	1.2089	-0.7746	1.1931	-1.0724
H	-0.5492	0.531	-1.3377	-0.1285	-1.5281	1.2651	-0.43	2.0477	0.4536

Table S8: Optimized XYZ coordinates for neutral EGDN conformer G at the B3LYP/6-31G(2df,p) level of theory

	Conf. G		
Atom	X	Y	Z
C	0.8153	-1.4835	-0.4213
C	-0.6419	-1.299	-0.0337
O	1.6837	-0.8129	0.5001
O	-0.962	0.0735	-0.3053
N	-2.2875	0.4303	0.0576
N	2.1815	0.4601	0.0603
O	-2.5254	1.585	-0.1504
O	-2.9868	-0.4443	0.5094
O	1.9481	0.7988	-1.0702
O	2.8012	1.009	0.9238
H	1.0083	-1.1399	-1.4379
H	1.0754	-2.5427	-0.3378
H	-0.7949	-1.519	1.0259
H	-1.2763	-1.963	-0.6299

Table S9: Optimized XYZ coordinates for cationic EGDN conformers I^+, II^+, and III^+ at the B3LYP/6-31G(2df,p) level of theory

	Conf. I^+			Conf. II^+			Conf. III^+		
Atom	X	Y	Z	X	Y	Z	X	Y	Z
C	0.7732	-1.4464	-0.5722	0.9316	-1.6681	-0.2195	-0.5752	-0.1566	0.7478
C	-0.6736	-1.4289	0.6062	-0.9316	-1.6681	0.2195	0.5752	0.1566	-0.7478
O	1.8393	-1.0229	0.0496	1.2187	-0.5922	-0.8906	-1.598	-0.8488	0.3377
O	-0.9781	-0.1945	0.9076	-1.2187	-0.5922	0.8906	1.598	0.8488	-0.3377
N	-2.2732	0.4529	-0.0242	-1.972	0.6344	-0.0675	2.9951	-0.106	0.0133
N	2.1172	0.6574	-0.1223	1.972	0.6344	0.0675	-2.9951	0.106	-0.0133
O	-2.6829	-0.319	-0.8059	-2.1415	1.5449	0.6357	3.8315	0.635	0.3293
O	-2.447	1.537	0.3562	-2.1464	0.2859	-1.1719	2.7941	-1.2503	-0.1443
O	1.318	1.17	-0.813	2.1464	0.2859	1.1719	-3.8318	-0.635	-0.3284
O	3.0648	0.9087	0.5006	2.1415	1.5449	-0.6357	-2.7938	1.2504	0.1435
H	0.381	-0.8097	-1.3725	0.9146	-2.5785	-0.8288	-0.7533	0.8814	1.0479
H	0.8022	-2.5235	-0.7715	1.3538	-1.7501	0.7879	0.1169	-0.7382	1.3635
H	-0.2177	-1.9722	1.4397	-0.9146	-2.5785	0.8289	-0.1169	0.7382	-1.3636
H	-1.4006	-1.9805	-0.0029	-1.3538	-1.7501	-0.7879	0.7533	-0.8814	-1.0479

Table S10: Optimized XYZ coordinates for cationic EGDN conformer IV^+ at the B3LYP/6-31G(2df,p) level of theory

	Conf. IV^+		
Atom	X	Y	Z
C	0.5933	-0.6907	-0.7323
C	-0.5933	-0.6904	0.7326
O	1.329	0.3845	-0.7231
O	-1.329	0.3848	0.7229
N	-2.8571	0.1783	-0.036
N	2.8571	0.1784	0.0359
O	-2.9937	-0.9057	-0.4647
O	-3.4162	1.192	0.0573
O	2.994	-0.9056	0.4646
O	3.416	1.1922	-0.0574
H	-0.1379	-0.6979	-1.5461
H	1.097	-1.6411	-0.5167
H	0.1379	-0.6972	1.5465
H	-1.097	-1.6409	0.5175

We then compared the RMSD values between the optimized ionized neutral conformers and the optimized cationic conformers to establish relationships between neutral and cationic geometries. We calculated the relaxation energies for each conformer, which represent the energy difference between the vertically ionized and fully relaxed cationic structures. The resulting mapping between neutral and cationic conformers, along with the associated relaxation energies, are shown in Figure S4. Boltzmann population analysis revealed that neutral conformers A and B represent $\sim 69\%$ of the total population at 298.15 K. Cation conformers I^+, II^+, and III^+ also have similar populations, and combined they amount to $\sim 86\%$ of the total population (Table S11).

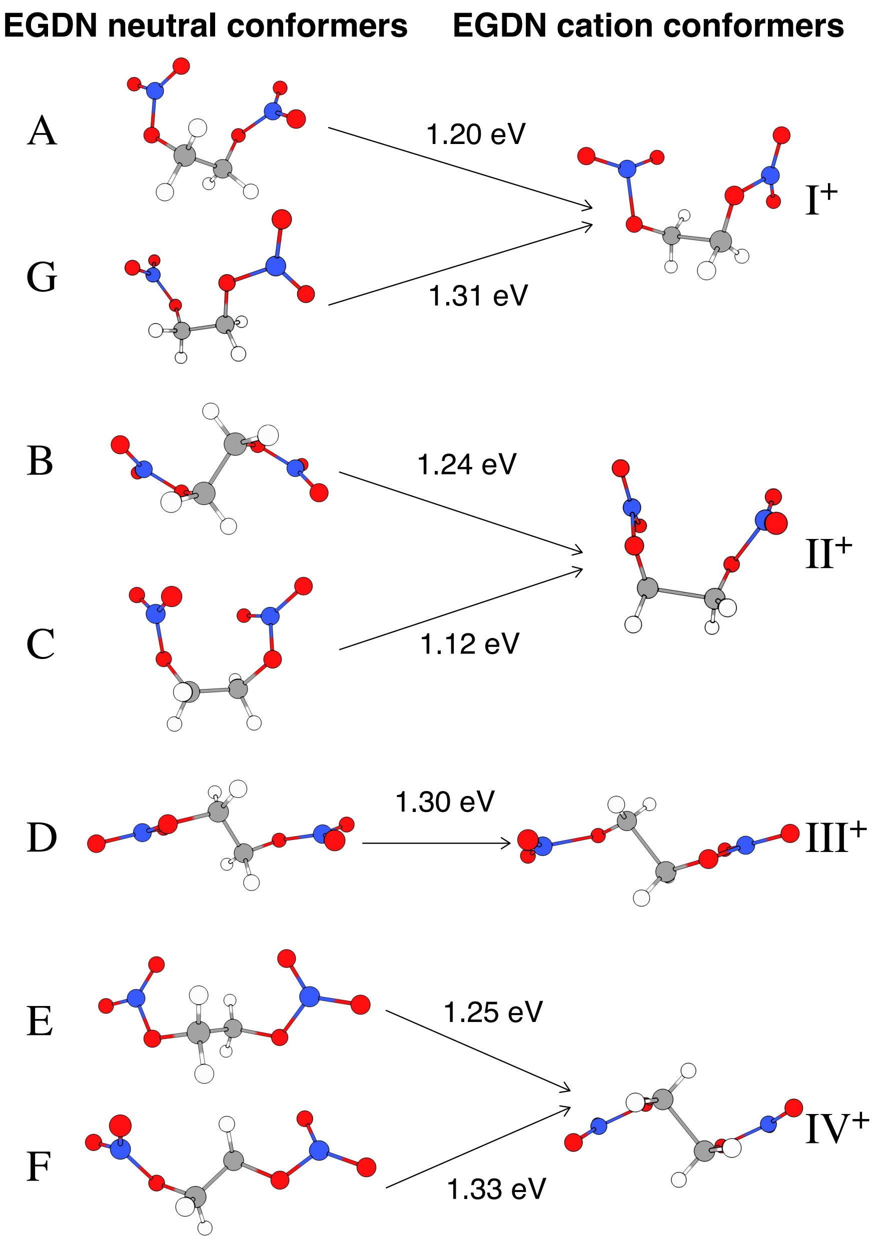

Figure S4: Proposed relationships between the 7 neutral and 4 cationic EGDN conformers, including their respective relaxation energies. The relaxation energy represents the energy difference between the vertically ionized and fully relaxed cationic structures for each conformer.

Table S11: Population analysis for EGDN neutral conformers (A-G) and cationic conformers (I^+-IV^+).

Conformer	% Population	Rel. energy (eV)	exp(-ΔE/RT)
A	32.3	9.7050	0.8844
B	36.6	9.7082	1.0000
C	2.7	9.6417	0.0751
D	9.9	9.6746	0.2703
E	5.8	9.6609	0.1590
F	5.5	9.6593	0.1492
G	7.2	9.6664	0.1965
I^+	31.2	9.6763	1.0000
II^+	26.4	9.7081	0.8468
III^+	28.1	9.6980	0.9033
IV^+	14.3	9.7083	0.4591

S2.2 EOM-CCSD calculations

Equation of motion-coupled cluster singles and doubles calculations (EOM-CCSD) was used to identify the excited state energies and corresponding oscillator strength for each transition. We first used EOM-CCSD calculations at the 6-311+G(d) level of theory to examine the excited states and transitions for neutral conformers B and G. Although conformer A has a higher population, our selection of conformers B and G was guided by both practical constraints and strategic considerations. First, the computational expense of each coupled cluster calculation necessitated a selective approach. More importantly, conformers B and G play unique roles in conformer mapping. As illustrated in Figure S4, these neutral conformers relax to two distinct cation conformers, allowing us to efficiently explore the conformational landscape and its impact on electronic transitions. Additionally, conformer G holds particular significance for our subsequent AIMD simulations because upon vertical ionization, the G^+_{vert} structure is a transition state that can be used to initiate AIMD simulations (see Section S2.4 for details). By focusing on conformers B and G, we strike a balance between exploring conformational diversity, managing computational resources, and setting the stage for subsequent dynamic simulations, while working within the constraints of our computa-

tional resources. Figure S5 and Table S12 depict the excited state energies and the oscillator strengths for the transitions from S_0 for neutral conformers B and G.

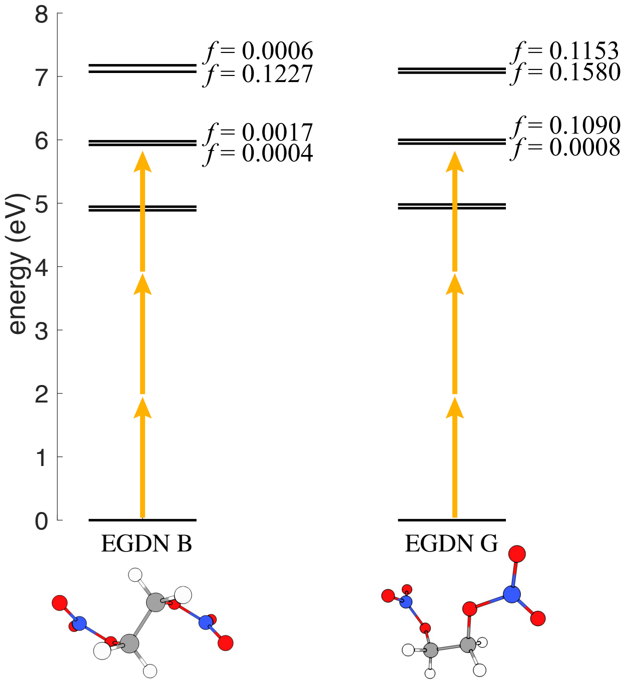

Figure S5: Electronic excited state energies and oscillator strength (f) of transitions out of the S_0 state for neutral conformer B (left) and neutral conformer G (right) at the EOM-CCSD/6-311+G(d) level of theory.

The neutral conformer B exhibits a particularly strong transition at 7.0741 eV with an oscillator strength of $f = 0.1227$ and a weak transition at 5.9598 eV with $f = 0.0017$. Other transitions have negligible oscillator strengths and therefore less likely to occur. The neutral conformer G has the strongest transitions at 7.0631 eV and 7.1217 eV, which are of similar magnitude to the strong transition in the B conformer. Additionally, the transition at 5.9950 eV is significant, with a much higher oscillator strength than the corresponding transition in the B conformer. These results were compared to the experimental UV-VIS spectra of EGDN as reported in ref. S25 (Figure S6). Our theoretical predictions of the bright S_4 – S_6 states having energies in the range of 6.0 – 7.1 eV show reasonable agreement with the experimental UV-VIS spectrum that exhibits a peak at approximately 188 nm (6.6 eV),[S25] providing support for the validity of our calculations. However, it's important to note po-

tential sources of discrepancies between theory and experiment. The experimental spectra were measured in methanol solution, while our calculations represent gas-phase conditions. Methanol, as a polar solvent, can interact with EGDN molecules, potentially shifting peak positions, altering intensities, or broadening spectral features. Additionally, limitations in our theoretical method may not fully account for all aspects of electron correlation. Despite these considerations, the qualitative agreement between our theoretical results and experimental data lends credibility to our approach.

Table S12: Excitation energies (EE) and oscillator strengths (f) of neutral conformers B and G calculated at the EOM-CCSD/6-311+G(d) level of theory.

**EGDN B**			
State	Wavelength (nm)	Excitation energy (eV)	oscillator strength (f)
S_1	251.70	4.9258	0.0000
S_2	251.63	4.9272	0.0001
S_3	208.08	5.9585	0.0004
S_4	208.03	5.9598	0.0017
S_5	175.27	7.0741	0.1227
S_6	172.77	7.1764	0.0006
**EGDN G**			
State	Wavelength (nm)	Excitation energy (eV)	oscillator strength (f)
S_1	251.05	4.9386	0.0000
S_2	250.18	4.9559	0.0007
S_3	208.77	5.9389	0.0008
S_4	206.81	5.9950	0.1090
S_5	175.54	7.0631	0.1581
S_6	174.09	7.1217	0.1153

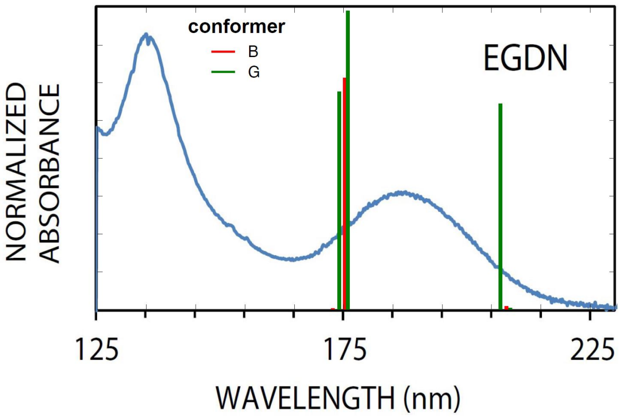

Figure S6: Experimental VUV-VIS spectra for EGDN in methanol (blue)[S25] compared to computed spectra of Neutral B (red) and G (green) at the EOM-CCSD level.

EOM-CCSD calculations were also conducted for the vertically ionized neutral B, B^+_{vert}, and cationic EGDN conformers I^+ and II^+. It's worth noting that similar calculations were attempted for $Gvert^+$, but these did not converge, likely due to the unique electronic structure or geometry of this vertically ionized state. Consequently, our analysis focuses solely on the results obtained for B^+_{vert}. Table S13 displays the energies and oscillator strengths for B^+_{vert} corresponding to the diagram shown in Figure 3 in the main text. The energies and oscillator strengths for the cation conformers I^+ and II^+ are shown in Figure S7 and Table S14. The energy gaps between D_0 and the excited states D_1 through D_5 are significantly lower for B^+_{vert} than for the optimized cation conformers I^+ and II^+. This increased energy requirement for electronic excitation in the cationic form is likely due to the structural relaxation that occurs after vertical ionization. In contrast, conformers I^+ and II^+ have similar excitation energies and oscillator strengths, indicating little difference in electronic structure between different cation conformers. Both structures would require absorption of two probe photons at 1.9 eV (orange arrows in Figure S7) to reach any excited state.

Table S13: Electronic excited state energies and corresponding oscillator strength (f) of transitions out of the D_0 state for B^+_{vert} at the EOM-CCSD/6-311+G(d) level of theory.

**EGDN $\mathbf{B^+_{vert}}$**			
State	Wavelength (nm)	Excitation energy (eV)	oscillator strength (f)
D_1	1278.4512	0.9698	0.0175
D_2	1054.1977	1.1761	0.0007
D_3	979.4154	1.2659	0.0048
D_4	841.4265	1.4735	0.0006
D_5	816.2762	1.5189	0.0971
D_6	486.6897	2.5475	0.0001
D_7	327.1264	3.7901	0.0095

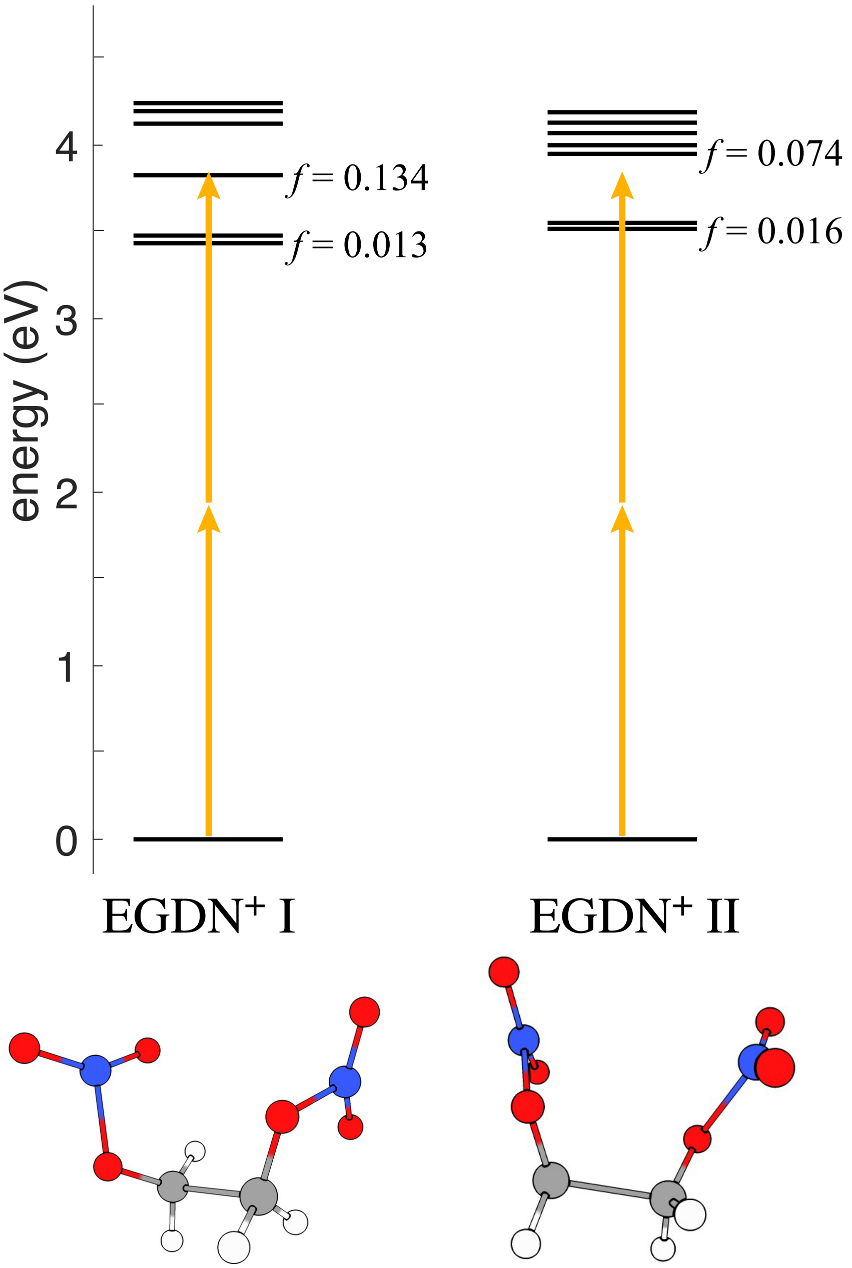

Figure S7: Electronic excitation energy states and oscillator strengths (f) for relaxed cationic conformer I^+ (left) and relaxed cationic conformer II^+ (right) at the EOM-CCSD/6-311+G(d) level of theory.

Table S14: Electronic excitation energies (EE) and corresponding oscillator strengths (f) for relaxed cationic conformers I^+ and II^+ at the EOM-CCSD/6-311+G(d) level of theory.

**EGDN I^+**			
State	Wavelength (nm)	Excitation energy (eV)	oscillator strength (f)
D_1	360.9123	3.4353	0.0135
D_2	356.3583	3.4792	0.0093
D_3	323.9554	3.8272	0.1336
D_4	300.6260	4.1242	0.0051
D_5	295.4537	4.1964	0.0032
D_6	292.2432	4.2425	0.0275
**EGDN II^+**			
State	Wavelength (nm)	Excitation energy (eV)	oscillator strength (f)
D_1	351.3594	3.5287	0.0163
D_2	350.4556	3.5378	0.0010
D_3	313.8443	3.9505	0.0738
D_4	310.0613	3.9987	0.0000
D_5	304.5920	4.0705	0.0145
D_6	300.0803	4.1317	0.0000

S2.3 Fragmentation pathway energetics

The energies associated with fragmentation pathways producing the observed ${CH_2NO_3}^+$, ${NO_2}^+$, and CH_2O^+ ions were calculated using DFT at the B3LYP/6-31G(2df,p) level of theory. These calculations determined the enthalpy required at 298.15 K to break a given bond of a specific molecular entity through homolysis. In this process, a single covalent bond is cleaved, with the bonding electrons equally distributed between the two resulting fragments.We first calculated the energy associated with the $O-NO_2$ bond in neutral EGDN conformer B to be 149.05 kJ/mol, in good agreement with the literature value of 140.46 kJ/mol.[S15,S20] It's worth noting that the literature value was obtained using the B3LYP/6-31G* level of theory, while our calculations employed the more constrained B3LYP/6-31G(2df,p) level. The robust agreement despite different basis sets supports the use of the B3LYP/6-31G(2df,p) level for subsequent calculations on BDE values in EGDN cation. The pathways producing the observed ${CH_2NO_3}^+$ (m/z 76), ${NO_2}^+$ (m/z 46), and CH_2O^+

(m/z 30) fragments for EGDN conformer I^+ are shown in Figure S8. We determined that the energy of each pathway was similar among all EGDN cation conformers I^+ – IV^+ (Table S15). The prevalence of NO_2 fragments and ${NO_2}^+$ ions having the lowest dissociation barrier also stands out in these calculations. It should be noted that these fragments are common for other explosives and are thought to correlate well to impact sensitivity.[S26,S27]

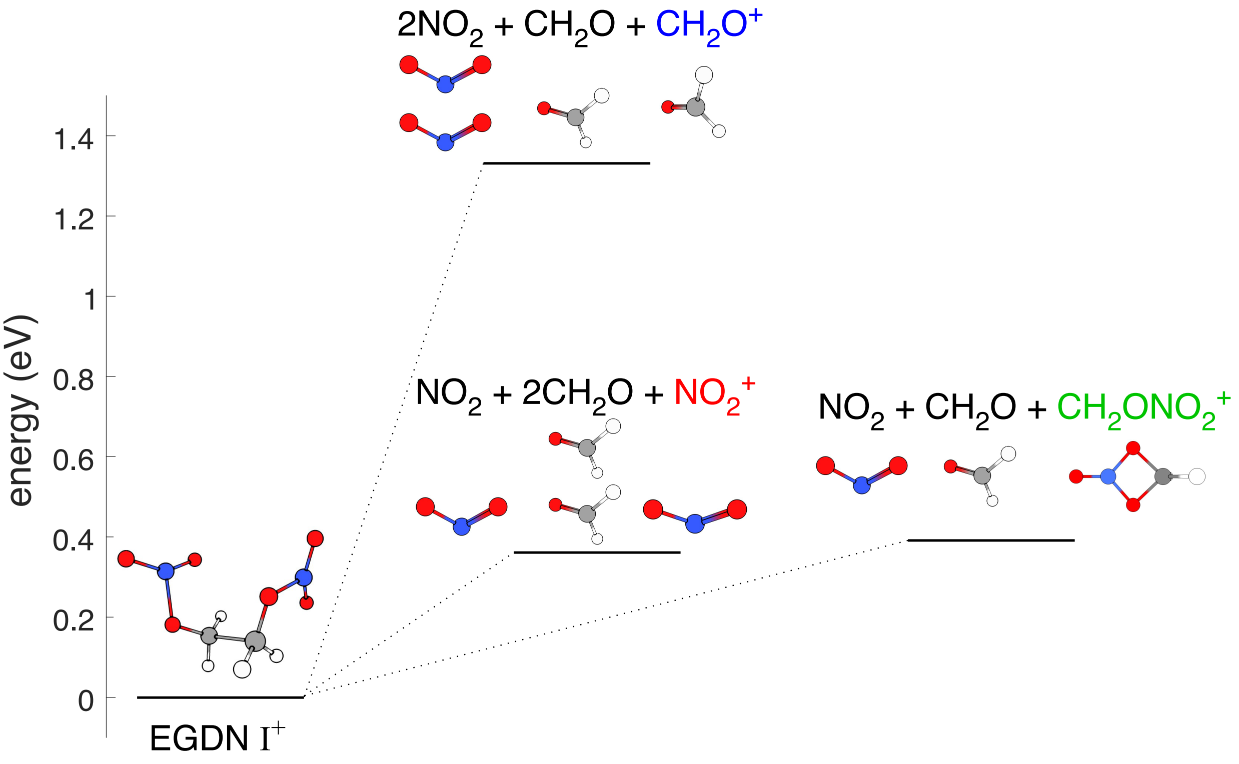

Figure S8: Computed dissociation pathways from EGDN I^+ at the B3LYP/6-31G(2df,p) level.

Table S15: Dissociation energies (eV) for EGDN cationic conformers I^+–IV^+ to produce the fragment ions m/z 76, 46, and 30 calculated at the B3LYP/6-31G(2df,p) level.

**Fragment ion**	**Conformer I^+**	**Conformer II^+**	**Conformer III^+**	**Conformer IV^+**
m/z 76	0.39	0.36	0.37	0.36
m/z 46	0.36	0.33	0.34	0.33
m/z 30	1.33	1.30	1.31	1.30

S2.4 *Ab Initio* Molecular Dynamics (AIMD)

Our AIMD simulations were designed to track the dynamics of EGDN cation following vertical ionization. First, frequency calculations were performed using Gaussian 16 on each neutral conformer (A–G) after vertical ionization. Of these conformers, only the vertically ionized structure of Conformer G (G^+_{vert}) had a single imaginary frequency (i.e., was a transition state); the remaining conformers A^+_{vert} – F^+_{vert} had two or more imaginary frequencies (Table S16).

Table S16: Frequency calculations completed on vertical cation conformers. Conformer G^+_{vert} had a single imaginary frequency (i.e., was a transition state); the remaining conformers A^+_{vert} – F^+_{vert} had two or more imaginary frequencies.

Conformer	Negative Frequencies (cm^{-1})
G^+_{vert}	−1549.15
A^+_{vert}	−1453.32, −775.59, −32.83
B^+_{vert}	−1466.60, −623.15
C^+_{vert}	−1163.83, −689.21
D^+_{vert}	−1467.28, −615.56, −15.67
E^+_{vert}	−1365.04, −566.88
F^+_{vert}	−1065.13, −289.46

Because AIMD simulations, as implemented in Q-Chem 5.3, can only be initiated from stationary points (local minima or transition states) on the potential energy surface, the G^+_{vert} structure was the only conformer that could be used to directly initiate AIMD simulations following vertical ionization. To consider additional conformational effects on $EGDN^+$ dynamics, simulations were also initiated from the cationic conformers I^+ through IV^+. To simulate the excess energy in these conformers left after geometric relaxation from the vertically ionized structures, the energy differences in Figure S4 were used, ranging from 1.12–1.33 eV, depending on the cation conformer. Our AIMD simulations localized this excess energy in the initial $EGDN^+$ structure without altering its nuclear geometry. This technique had previously been used to add excess energy to simulate ionization into an electronically excited state in nitromethane.[S18] As the simulation progresses, this excess energy redistributes from the electronic structure to various nuclear coordinates and vibrational modes. Although this

redistribution tends to favor modes associated with bond breakage, it is not confined to a single bond or mode. The AIMD simulations capture this time-dependent energy redistribution process, revealing how the system evolves from its initial ionized state through bond breaking to eventual fragmentation.

80 trajectories were simulated for each conformer, with dynamics propagated in steps of 0.1 fs for a simulation time of approximately 1100 fs. This specific duration was chosen as a balance between computational feasibility and capturing relevant chemical dynamics. The 1100 fs time frame is expected to capture initial fragmentation events based on the T_1 and T_2 experimental time scales of less than 500 fs, while managing computational resources effectively. Longer simulations would have significantly increased computational costs and data storage requirements without necessarily providing additional insights into the primary dissociation pathways.

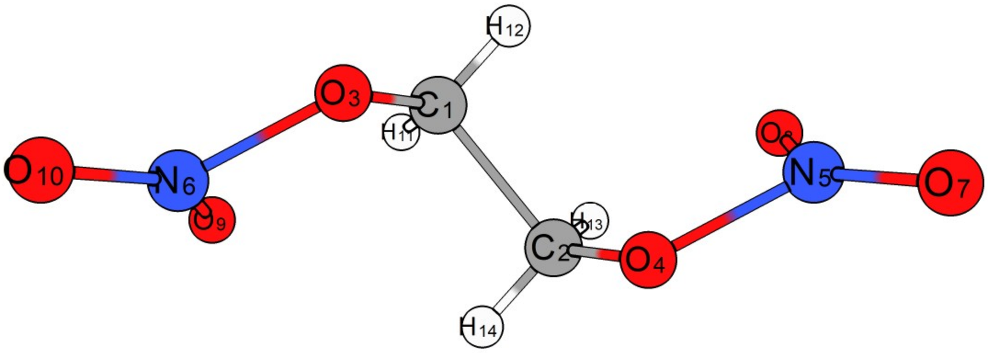

Figure S9: Structural representation of an EGDN conformer with atom labels. Carbon atoms are labeled as C_1 and C_2; oxygen atoms as O_1 through O_6; nitrogen atoms as N_1 and N_2; and hydrogen atoms as H_1 through H_4.

To determine the bond length threshold, we analyzed three key bonds across various cationic conformers of EGDN: the central C_1–C_2 bond connecting the two CH_2NO_3 groups, and the two O−N bonds (O_3-N_6 and O_4-N_5) linking the nitro groups to the carbon backbone (Figure S9). These bonds were chosen as they are critical to the molecule's structural integrity and are likely to be involved in the initial fragmentation process. We calculated the average bond length and standard deviation for each bond type. The breakage threshold for

each bond was then set at three standard deviations above its average length. This statistical approach ensures that the threshold captures significant bond elongations while excluding normal thermal fluctuations. The C_1–C_2 bond threshold was set at 2.841 Å, representing a 24% increase from its average length of 1.894 Å. Similarly, the O_3-N_6 and O_4-N_5 bonds had thresholds of 2.585 Å and 2.579 Å, respectively, corresponding to approximately 20% increases from their average lengths. This method provides a robust criterion for identifying the onset of fragmentation, as it typically encompasses 99.7% of all data points in a normal distribution, effectively distinguishing between intact and breaking bonds.

Using these bond-breaking criteria, we defined the initial fragmentation time (IFT) as the time of the first bond-breaking event in the trajectory. The statistical distribution of IFT values for each initial conformer are given in Table S17. The corresponding distributions of IFT values are detailed in Figure S10.

Table S17: Mean and Standard Deviation IFT values for Cationic EGDN Conformers I^+–IV^+ and conformer ${G_{vert}}^+$

Conformer	IFT (fs)
I^+	647 ± 293
II^+	307 ± 200
III^+	478 ± 263
IV^+	533 ± 42
${G_{vert}}^+$	118 ± 104

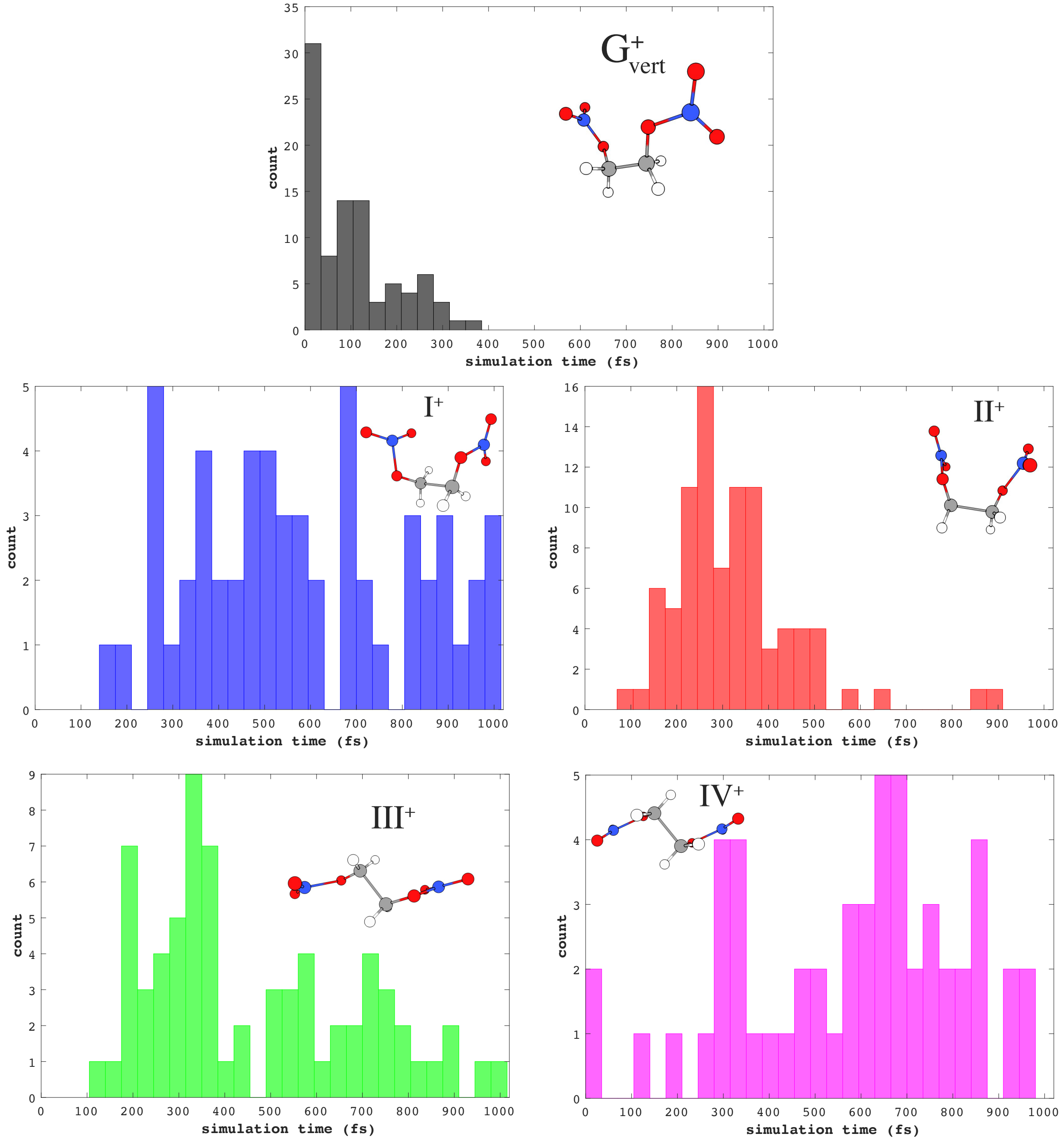

Figure S10: Comparison of IFT distributions for EGDN cationic conformers I^{+}–IV^{+} and $G_{vert}{}^{+}$.

To further validate our AIMD analysis, we employed additional statistical chi-square (χ^2) and t-tests. These tests provide a rigorous framework for comparing the dissociation behaviors of different conformers. The t-test was used to determine whether there is a

significant difference between the IFT distributions for different pairs of conformers. Table S18 shows the t-statistics and associated p values obtained for each pair of conformers, with $p < 0.001$ values indicating a statistically significant difference between the IFT distributions of the pair.

Table S18: T-test Statistics and p-values for EGDN Conformers

		$\mathbf{II^+}$	$\mathbf{III^+}$	$\mathbf{IV^+}$	$\mathbf{G_{vert}}^+$
**t-Statistic**	$\mathbf{I^+}$	8.3927	1.2142	0.20944	-13.8865
**p-value**		< 0.001	0.227	0.8341	< 0.001
**t-Statistic**	$\mathbf{II^+}$		-4.4994	-6.0134	13.8865
**p-value**			< 0.001	< 0.001	< 0.001
**t-Statistic**	$\mathbf{III^+}$			-0.9731	15.1461
**p-value**				0.3319	< 0.001
**t-Statistic**	$\mathbf{IV^+}$				10.1466
**p-value**					< 0.001

The chi-square statistic was calculated based on the probabilities of breaking at least one bond within the 1100 fs simulation time. It quantifies the deviation between observed and expected probabilities of breaks, assuming the null hypothesis of no difference between conformer pairs. Conformer pairs obtaining $p < 0.001$ indicate a significant difference in bond-breaking probability (Table S19).

Table S19: chi-square (χ^2) statistics and p-values for pairs of EGDN conformers. The statistic was not calculated for the pair of G^+_{vert} and II^+ because 100% of trajectories in both conformers dissociated.

		$\mathbf{II^+}$	$\mathbf{III^+}$	$\mathbf{IV^+}$	$\mathbf{G_{vert}}^+$
χ^2	$\mathbf{I^+}$	34.0308	0.0181	0.0279	34.0308
**p-value**		< 0.001	0.8931	0.5209	< 0.001
χ^2	$\mathbf{II^+}$		35.0010	29.6992	–
**p-value**			< 0.001	< 0.001	–
χ^2	$\mathbf{III^+}$			0.399	12.986
**p-value**				0.528	< 0.001
χ^2	$\mathbf{IV^+}$				29.699
**p-value**					< 0.001

Interpretation of these statistics reveals three distinct groups of conformers:

- Group 1: Conformers I^+, III^+, and IV^+. This group has significantly greater IFT values than either II^+ or G^+_{vert} and is the only group with nonzero probability of remaining intact after the 1100 fs simulation window.
- Group 2: Conformer II^+, with a significantly shorter IFT than the conformers in Group 1, but significantly longer IFT than G^+_{vert}
- Group 3: G^+_{vert}, with the shortest IFT.

References

[S1] Fettaka, H.; Lefebvre, M. Ethylene Glycol Dinitrate (EGDN): from Commercial Precursors, Physicochemical and Detonation Characterization. *Central European Journal of Energetic Materials* **2015**, *12*, 287–305.

[S2] http://webbook.nist.gov/chemistry/. Last checked 10/24/2024.

[S3] Ampadu Boateng, D.; Word, M. D.; Gutsev, L. G.; Jena, P.; Tibbetts, K. M. Conserved Vibrational Coherence in the Ultrafast Rearrangement of 2-Nitrotoluene Radical Cation. *J. Phys. Chem. A* **2019**, *123*, 1140–1152.

[S4] Keldysh, L. Ionization in Field of a Strong Electromagnetic Wave. *Sov. Phys. JETP* **1965**, *20*, 1307–1314.

[S5] Topcu, T.; Robicheaux, F. Dichotomy between tunneling and multiphoton ionization in atomic photoionization: Keldysh parameter γ versus scaled frequency Ω. *Phys. Rev. A* **2012**, *86*, 053407.

[S6] Jochim, B.; DeJesus, L.; Dantus, M. Ultrafast Disruptive Probing: Simultaneously Keeping Track of Tens of Reaction Pathways. *Rev. Sci. Instrum.* **2022**, *93*, 033003.

[S7] Lucchini, M.; Mignolet, B.; Murari, M.; Gonçalves, C. E. M.; Lucarelli, G. D.; Frassetto, F.; Poletto, L.; Remacle, F.; Nisoli, M. Few-Femtosecond C2H4+ Internal Relaxation Dynamics Accessed by Selective Excitation. *J. Phys. Chem. Lett.* **2022**, *13*, 11169–11175.

[S8] Motulsky, H. J.; Ransnas, L. A. Fitting Curves to Data Using Nonlinear Regression: a Practical and Nonmathematical Review. *The FASEB Journal* **1987**, *1*, 365–374.

[S9] Grimme, S. Exploration of Chemical Compound, Conformer, and Reaction Space with Meta-Dynamics Simulations Based on Tight-Binding Quantum Chemical Calculations. *Journal of Chemical Theory and Computation* **2019**, *15*, 2847–2862.

[S10] Pracht, P.; Bohle, F.; Grimme, S. Automated exploration of the low-energy chemical space with fast quantum chemical methods. *Phys. Chem. Chem. Phys.* **2020**, *22*, 7169–7192.

[S11] Grimme-Lab GitHub - grimme-lab/crest: Conformer-Rotamer Ensemble Sampling Tool based on the xtb Semiempirical Extended Tight-Binding Program Package. `https://github.com/grimme-lab/crest`.

[S12] Grimme-Lab GitHub - grimme-lab/xtb: Semiempirical Extended Tight-Binding Program Package. `https://github.com/grimme-lab/xtb`.

[S13] Bannwarth, C.; Caldeweyher, E.; Ehlert, S.; Hansen, A.; Pracht, P.; Seibert, J.; Spicher, S.; Grimme, S. Extended tight-binding quantum chemistry methods. *WIREs Computational Molecular Science* **2021**, *11*, e1493.

[S14] Frisch, M.; Trucks, G.; Schlegel, H.; Scuseria, G.; Robb, M.; Cheeseman, J.; Scalmani, G.; Barone, V.; Mennucci, B.; Petersson, G.; et al *Gaussian 16 Rev. B.01.* Gaussian, Inc. Wallingford, CT, 2016.

[S15] Li, M.; Guo, X.; Li, F.; Song, H. Theoretical Studies on the Structures, Thermodynamic Properties, Detonation Performance, and Pyrolysis Mechanisms for Six Dinitrate Esters. *Chinese Journal of Chemistry* **2009**, *27*, 1871–1878.

[S16] Karton, A.; Martin, J. M. L. Explicitly correlated Wn theory: W1-F12 and W2-F12. *The Journal of Chemical Physics* **2012**, *136*, 124114.

[S17] Bursch, M.; Mewes, J.-M.; Hansen, A.; Grimme, S. Best-Practice DFT Protocols for Basic Molecular Computational Chemistry. *Angewandte Chemie International Edition* **2022**, *61*, e202205735.

[S18] Word, M. D.; López Peña, H. A.; Ampadu Boateng, D.; McPherson, S. L.; Gutsev, G. L.; Gutsev, L. G.; Lao, K. U.; Tibbetts, K. M. Ultrafast Dynamics of Nitro–Nitrite Rearrangement and Dissociation in Nitromethane Cation. *J. Phys. Chem. A* **2022**, *126*, 879–888.

[S19] Gong, X.; Xiao, H. Studies on the molecular structures, vibrational spectra and thermodynamic properties of organic nitrates using density functional theory and ab initio methods. *Journal of Molecular Structure: THEOCHEM* **2001**, *572*, 213–221.

[S20] Zeng, X.-L.; Chen, W.-H.; Liu, J.-C.; Kan, J.-L. A theoretical study of five nitrates: Electronic structure and bond dissociation energies. *Journal of Molecular Structure: THEOCHEM* **2007**, *810*, 47–51.

[S21] Curtiss, L. A.; Redfern, P. C.; Raghavachari, K. Gaussian-4 theory. *The Journal of Chemical Physics* **2007**, *126*, 084108.

[S22] Gutsev, G. L.; McPherson, S. L.; López Peña, H. A.; Boateng, D. A.; Gutsev, L. G.; Ramachandran, B. R.; Tibbetts, K. M. Dissociation of Singly and Multiply Charged Nitromethane Cations: Femtosecond Laser Mass Spectrometry and Theoretical Modeling. *J. Phys. Chem. A* **2020**, *124*, 7427–7438.

[S23] Türker, L.; Şakir Erkoç Density functional theory calculations for [C2H4N2O6](n) (n=0, +1, -1). *Journal of Hazardous Materials* **2006**, *136*, 164–169.

[S24] Türker, L. Quantum chemical studies on EGDN and its monovalent ions. *Journal of Molecular Structure: THEOCHEM* **2005**, *717*, 9–14.

[S25] Cruse, C. A.; Goodpaster, J. V. Generating highly specific spectra and identifying thermal decomposition products via Gas Chromatography / Vacuum Ultraviolet Spectroscopy (GC/VUV): Application to nitrate ester explosives. *Talanta* **2019**, *195*, 580–586.

[S26] Keshavarz, M. H. Simple Relationship for Predicting Impact Sensitivity of Nitroaromatics, Nitramines, and Nitroaliphatics. *Propellants, Explosives, Pyrotechnics* **2010**, *35*, 175–181.

[S27] Ye, S.; Tonokura, K.; Koshi, M. Energy transfer rates and impact sensitivities of crystalline explosives. *Combustion and Flame* **2003**, *132*, 240–246.
